# Fight, retreat, repeat: The male–male agonistic behavior in the wood‐feeding cockroach, *Panesthia angustipennis spadica* (Dictyoptera: Blattodea: Blaberidae)

**DOI:** 10.1002/ece3.70319

**Published:** 2024-10-17

**Authors:** Haruka Osaki, Tomohiro Nakazono, Kiyotaka Yabe, Mamoru Takata, Aram Mikaelyan

**Affiliations:** ^1^ Department of Entomology and Plant Pathology North Carolina State University Raleigh North Carolina USA; ^2^ Laboratory of Insect Ecology, Graduate School of Agriculture Kyoto University Sakyo‐ku Kyoto Japan

**Keywords:** competition, field observation, fighting experience, male–male agonistic behavior, *Panesthia*, wood‐feeding cockroach

## Abstract

Competition is one of the most critical factors affecting animal behaviors. Aggressive interactions are central to acquiring resources or mating partners. Agonistic behavior is more common among males than females. Although laboratory observations of these behaviors give detailed descriptions under controlled conditions, field observations without human intervention are required because those supply information that provides insights into their function. In this paper, we report on the field observation and auxiliary laboratory experiments of male–male agonistic behavior of a wood‐feeding cockroach, *Panesthia angustipennis*, and discuss its strategy. In the field, a male pushed the opponent with the horn on the pronotum out of a gap between two logs, under which a female was. After pushing, the male repeatedly returned to a place close to the female, even if it did not subdue the opponent entirely. It suggests that the male–male agonistic behavior in *P. angustipennis* has both attack and avoidance. The bout was repeated as the ejected male reapproached the male. In contrast, the inferior male often escaped in the laboratory recording after field observation. Keeping the fighting experience for several days may contribute to the males avoiding a “losing battle.” This study significantly enhances our understanding of the mating strategy of *P. angustipennis* through male–male agonistic behavior and provides possibilities for its cognitive aspects from the fighting experience.

## Graphical abstract

It is the report of the wild male–male agonistic behavior in *Panesthia angustipennis*. Its fighting has remarkable characteristics, both aggressiveness and avoidance.

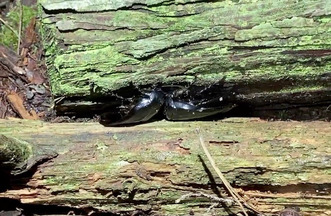

## 1 INTRODUCTION

Competition is a fundamental driving force shaping both survival and reproductive strategies in animals. Aggressive behavior, particularly male–male agonistic behavior, is a universal phenomenon reported across diverse organisms (Pandolfi et al., 2021). Notable examples of agonistic interactions aimed at securing females include mate guarding observed in crustaceans (Jormalainen, 1998) and harem defense in elephant seals (McCann, 1981). In addition, this behavior can involve defending the females from rival males even after mating (Kvarnemo, 2005; Suzuki, 1999). The outcomes of such male–male conflicts play a critical role in shaping the individual fitness and overall population dynamics (Andersson & Iwasa, 1996).

In this regard, *Panesthia angustipennis*, a wood‐feeding cockroach, presents a unique case study. *Panesthia angustipennis* is active outside the log at night during the breeding season, tunnels into rotten logs (Asahina, 1991), and exhibits ovoviviparity with significant gestation periods. Given the skewed field sex ratio indicating shorter adult longevity in males, male–male agonistic behavior in *P. angustipennis* has been anticipated (Ito & Osawa, 2019). Field observations are essential because natural male–male agonistic behavior may provide social information necessary to reveal its ecological dynamics. To date, however, there has been no report since the field observations of this behavior are challenging, given their nocturnal activity and low density, e.g., 2.07 adults/ha (Ito & Osawa, 2019).

In this paper, we report the wild male–male agonistic behavior of *P. angustipennis*, presenting both field and laboratory observations that illuminate not only the behavioral dynamics but also its social context. This study significantly enhances our understanding of male–male agonistic behavior and provides insight into this species’ cognitive ability for the fighting experience.

## 2 MATERIALS AND METHODS

### 2.1 Field observation and collection

On August 26, 2022, we eventually observed two males engaged in a struggle on two logs in Kyoto, Japan (34°52′36.9″ N, 135°51′59.3″ E) at 7:21 p.m. The event was documented on video using a smartphone (iPhone SE; Apple, Cupertino, US) with headlights (GH‐100RG; Gentos, Tokyo). The recording was terminated after 1:33 min into the struggle due to limitations imposed by the equipment. The two males, along with a female, were brought back to the laboratory, and the pronotum widths were measured. All three individuals were adults and had wings. The males were distinguished by their wings: Male X had torn wings, and Male Y had intact wings.

### 2.2 Laboratory observations

On the day following the collection, we conducted a series of experiments with the two males and the female. Our observations were recorded using a video camera (HC‐VX992MS; Panasonic, Tokyo, Japan) in controlled arenas (W200 × D150 × H100 mm). The experimental procedures were: (i) Male Y was introduced into the arena with Male X and the female. (ii) Both males were introduced simultaneously without the female. (iii) Male X was introduced into the arena with Male Y and the female. In treatments involving the female, (i and iii), a Petri dish (*φ*90 × 150 mm) with an entrance was used to ensure proximity between the males and the female at the beginning of the introduction. The female and male entered the petri dish 10 min before introducing the other male. Although, in treatment (iii), the pair exited from the petri dish for 10 min, the introduction was conducted because the female and male were close to each other. All video recordings were terminated 1 min after the males had ceased their physical encounter. Following each test, the individuals were isolated for 24 h.

### 2.3 Statistical analysis

All behaviors were analyzed using Boris (version 7.9.7; (Friard & Gamba, 2016)). We compared the observed behaviors with the following behaviors, which were the ones of another species in the same genus, *Panesthia cribrata*, reported in laboratory observation by O'Neill et al. (1987): (i) *Push*: lowering their pronota by pulling their heads down toward their legs. (ii) *Pulse*: extending and contracting the abdomen. (iii) *Block*: turning away from its opponent and lowering the side of the body or tip of the abdomen facing its opponent. (iv) *Submission*: standing still, abdomen downturned at the tip, and antennae motionless. If the behaviors not reported were observed, we record them as new behaviors. Statistical analysis was conducted with R version 4.3.2 (R Core Team, 2023). We conducted the Friedman test to compare the behavioral counts demonstrated by each male across treatments. We were also interested in the difference between the two males in the number of counts of the behaviors when data were summed across treatments. Fisher's exact test was used to examine the independence of those behaviors.

## 3 RESULTS

### 3.1 Pronotum widths

**FIGURE 1:**
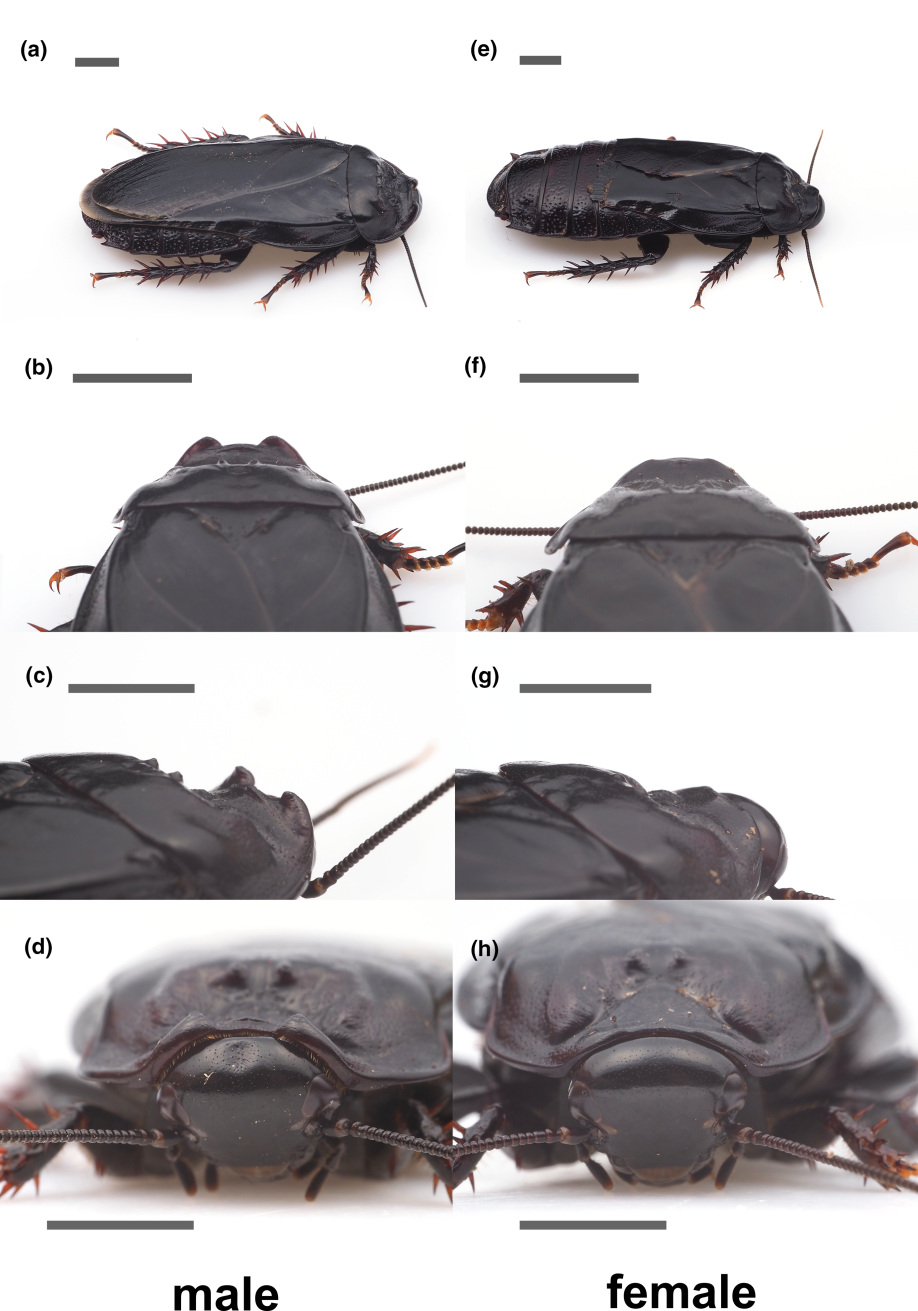
The photos on the left side are male and the right are female. (a) adult male, (b) male pronotum from the back side, (c) male pronotum from another angle, (d) male pronotum edge facing the head, (e) adult female, (f) female pronotum from the back side (g) female pronotum from another angle, and (h) female pronotum edge facing the head from the front. Only males have the horns on their pronotum. The scale bars indicate 5 mm.

The pronotum widths of the female, Male X and Male Y, were measured at 11.5, 13.5, and 13.1 mm, respectively. *Panesthia angustipennis* has a sexual dimorphism at the horns on the pronotum (Figure 1).

### 3.2 Field observation

**FIGURE 2:**
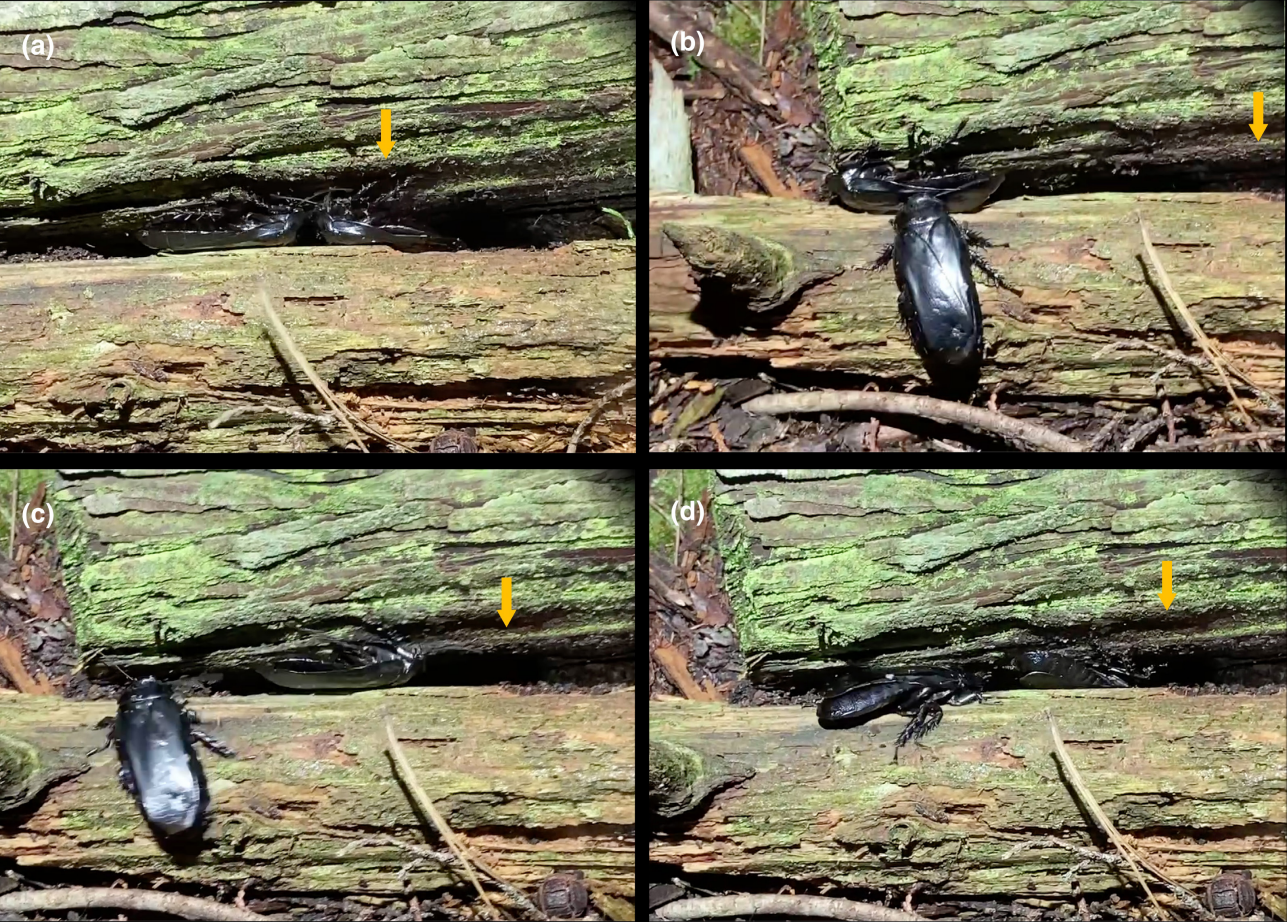
The behaviors observed during the male–male agonistic behavior. The arrow indicates the location of the home position. Although the arrow seems to move because of the camerawork, these four arrows indicate the same place. (a) The two males are pushing each other. (b) The lower male is retreating. (c) The right male is returning. (d) The left male is pursuing the opponent.

The digital video image is available in Video 1. Among the four behaviors that had been reported, we observed two: *push* (Figure 2a) and *block*. The newly observed behaviors are discussed in the following subsections.

**VIDEO 1:** The male–male agonistic behavior of *Panesthia angustipennis* in the field.

*Retreat*. A reaction in one male to being *pushed* or approached by another male, in which the male backs away without pivoting, often by evading any bout with the opponent (Figure 2b).

*Return*. The male pivots and withdraws from the other male after a bout, consistently to the same location in the arena defined as “home position,” which is situated directly above the female's location (Figure 2c).

*Pursue*. One male pursues the other male who is *returning* (Figure 2d).

*Stop*. A male ceases movement altogether.

The individual interactions observed as part of this agonistic behavior are as follows:

1. Initiation of the agonism with one male *pushing* the other.
2. A reciprocal *pushing* interaction, where both males engage in *pushing*.
3. Male X disengages and *returns* to the home position location within the arena.
4. Male Y *pursues* Male X again.
5. The sequence of interactions repeats through steps 1–4.

**FIGURE 3:**
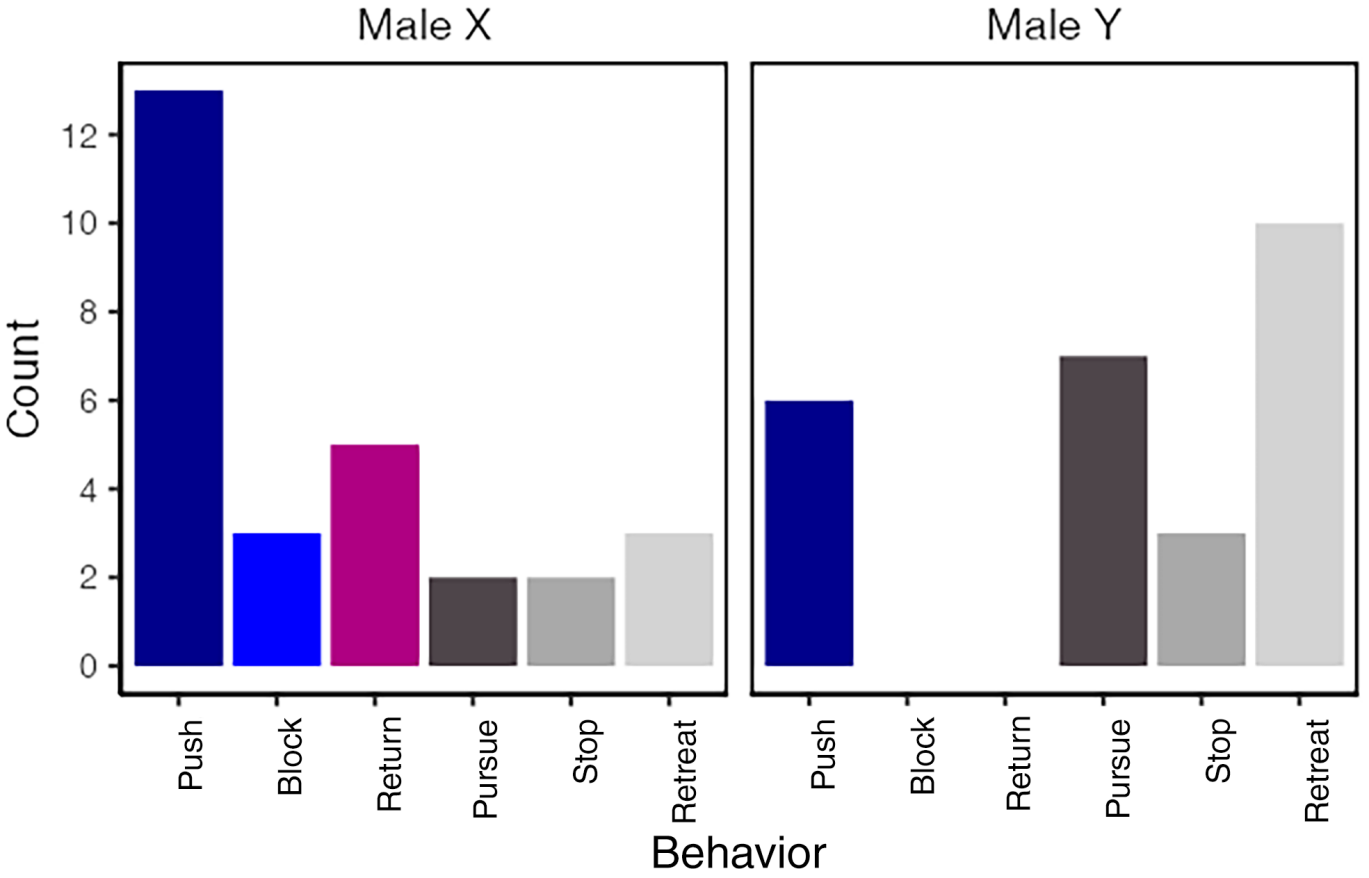
The number of each behavior recorded for Male X and Y in field observation.

Male X demonstrated both *block* and *return* behaviors, which were not exhibited by Male Y (Figure 3). Specifically, Male X displayed a high frequency of *push* and a moderate occurrence of *return*, while Male Y primarily showed *retreat*.

This pattern of bout was observed to repeat 16 times, predominantly initiated by Male X (13 times), and less frequently, Male Y (3 times). Specific to Male X were the *return* and *block* behaviors; the *return* was documented five times, which included *return* once each at 3, 7, and 15 cm from home position, and twice at 10 cm from the home position. The male *returned* at each place and went to home position. *Block* occurred three times, with two instances at 1 cm and one at 2 cm from home position. The female was not captured on video but remained stationary in the gap between two logs during the aggression bout.

None of the males' bouts involved the female. After observation, we found her under the log. The female remained absent from the fighting.

### 3.3 Laboratory observations

**FIGURE 4:**
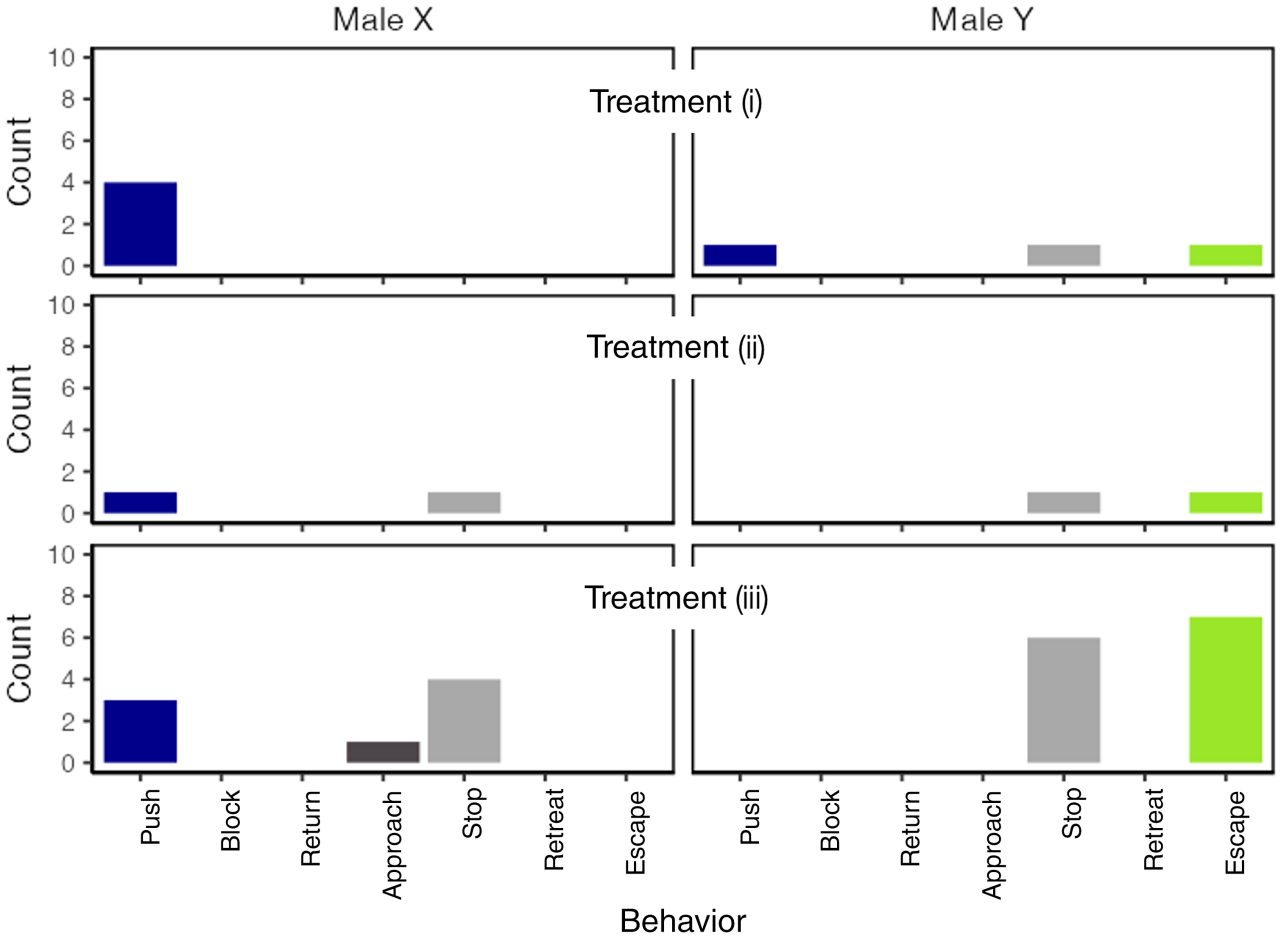
The number of each behavior responding to different treatments for Male X and Y in laboratory observation.

Recording times of treatment (i), (ii), and (iii) were 27, 6, and 59 min, respectively. Notably, in all treatments, Male Y ran away from Male X, which involved the male running in the opposite direction of their fighting partners. This behavior was named *escape* and observed in Male Y in all treatments (Figure 4).

There were no significant differences in the number of times of behaviors demonstrated by each male across treatments (Friedman test, Friedman *χ* ^2^ = 4.3, df = 2, *p* = .12). However, a significant difference was observed between the two males in the number of the *push* and *escape*: Male X engaged more frequently in *pushes* while Male Y engaged in *escapes* (Fisher's exact test, *p* < .05).

The observed sequence of the agonistic behavior remained consistent, regardless of the female's presence.

1. Initiation to *antennate* by one male.
2. The initiating male *pushes* the other male.
3. Male Y executes an *escape*.
4. Male X, often moving with heightened activity, would *antennate* with Male Y.
5. The sequence of interactions repeats through steps 1–4.

Agonistic behavior was initiated upon antennating, leading to one male *pushing* the other. Regardless of which of the males initiated the interaction, Male Y consistently *escaped*. Even upon *escape*, when the struggle between the males was staged again, Male Y's response to Male Xvs *push* was always to *escape* rather than to *push*.

The female did not join the fighting. Throughout the observation of these struggles, the female exhibited a certain posture, with her legs retracted and her head tucked under the pronotum.

## 4 DISCUSSION

The repeated *push* and *return* behavior in fighting, particularly the *return* of the male to a home position near the female, suggests two possibilities: enhancement of *return* by the gap between logs, and minimum aggression strategy. The gap may be easier to defend from within. To take this advantage, the male can be led to *return*. However, his attempt did not function well in displacing the opponent. In other words, it let the opponent pursue and challenge him immediately. To understand this inconsistency, we should also consider the benefit of quitting *pushing* in the middle of each struggle. This avoidance may reflect the strategy of *P. angustipennis* to maximize reproductive success while minimizing the costs associated with prolonged competition. Moreover, Male Y frequently *escaped* without severe fighting for 3 days, regardless of the treatments in the laboratory. This recurrence may contribute to avoiding extra fighting with the same opponent and suggest their recognition and retention.

The range defended by the male was approximately only twice the length of its body (around 10 cm). This territorial behavior indicates that the male tends to stay close to the resources: mate and a particular site. *Panesthia* is one of the closest genera to the subsocial wood‐feeding cockroach, genus *Salganea* (Djernæs & Varadinova, 2020), which is known for its biparental family structures (Maekawa et al., 2008). The *return* to the female is consistent with the hypothesis that the protection by males contributed to the evolution of biparental care (Royle et al., 2016).

A male pushed out of the home position would continue fighting. This male showed his persistence by repeating *pursue*. The repeated attempts to initiate struggles, even after expulsion, were another characteristic of the agonistic behavior in *P. angustipennis* other than *return*. Generally, avoiding more fighting than necessary can reduce energy costs and maintain higher expectations of reproductive success (Arnott & Elwood, 2008; Smith & Price, 1973). The aggressive behavior correlates to resource value (Enquist & Leimar, 1987; Liu & Hao, 2019). One of the possible reasons is the relatively short lifespan of *Panesthia* males, which are considered to have higher predation risk predicted by their behavioral tendencies (Ito & Osawa, 2019; O'Neill et al., 1987). Because of their shorter longevity, males might have limited opportunities to find females in their reproductive period. Another possible reason for repetition is their body sizes. The persistent struggle between the males could be attributed to the similarity in their body sizes, which likely complicates the establishment of dominance. This phenomenon has been noted in honeyeaters and spiders, wherein slight differences in body size can intensify or prolong the struggles (Austad, 1983; Kojima & Lin, 2017).

The *block*, observed only in Male X as well as *push* and particularly within close proximity to the home position, highlights its tactic using two different types of defense. The *block* has been described as an effective defense only in a small gallery by laboratory observation (O'Neill et al., 1987). However, we observed *blocks* outside galleries despite less effectiveness. The *block* requires no changing the position differently from the push, which requires turning the head to the other male. The male was considered to choose *block* as an instantaneous option in case the other male got too close toward the home position to *push* it.

They *pushed* each other using the pronotum, which displays sexual dimorphism with more pronounced horns in males. The horns were thought to serve as a weapon in *P. angustipennis*. Pronotums in other blaberid cockroaches, such as hissing cockroaches (Durrant et al., 2016), share this physical characteristic, further underscoring their importance in sexual selection and conflict resolution.

There is a strong need for more controlled laboratory experiments to explore further the triggers and consequences of the behaviors we observed in the field. Although we tried to observe those behaviors using the same individuals also in the laboratory, the *return* was unable to be recorded. Furthermore, Male Y did not engage in fighting and consistently escaped from Male X over 3 days. Its avoidance is consistent with the general theory of the contest behavior that losing decreases the willingness to fight (Hsu et al., 2005). Therefore, The initial interaction appears to have established Male X as the dominant individual, a status retained for at least 3 days. The recurrence of this outcome indicates a mechanism of status recognition and retention that warrants further investigation.

Our study suggests that antagonistic interactions in the males of *P. angustipennis* are composed of both engaging in and quitting fighting. It highlights the “strategic return” in the middle of the struggles. Moreover, retaining the fighting experience in *P. angustipennis* may contribute to avoiding fighting. We believe that our results contribute to understanding this species’ mating strategy and cognitive abilities. The information on these strategy and abilities may support the future exploring the evolution of biparental care in this group.

## AUTHOR CONTRIBUTIONS

**Haruka Osaki:** Conceptualization (lead); data curation (lead); formal analysis (lead); funding acquisition (equal); investigation (equal); methodology (lead); project administration (lead); resources (equal); software (lead); supervision (lead); validation (lead); visualization (lead); writing – original draft (lead); writing – review and editing (equal). **Tomohiro Nakazono:** Data curation (supporting); investigation (lead); resources (supporting); writing – review and editing (supporting). **Kiyotaka Yabe:** Data curation (supporting); investigation (lead); resources (supporting); writing – review and editing (supporting). **Mamoru Takata:** Data curation (supporting); funding acquisition (equal); investigation (lead); resources (supporting); writing – review and editing (supporting). **Aram Mikaelyan:** Conceptualization (equal); funding acquisition (equal); validation (supporting); writing – review and editing (equal).

## Data Availability

The data and R script can be found in the following URL: https://doi.org/10.7910/DVN/GFMOWM.

## References

[ece370319-bib-0001] Andersson, M. , & Iwasa, Y. (1996). Sexual selection. Trends in Ecology & Evolution, 11(2), 53–58. 21237761 10.1016/0169-5347(96)81042-1

[ece370319-bib-0002] Arnott, G. , & Elwood, R. W. (2008). Information gathering and decision making about resource value in animal contests. Animal Behaviour, 76(3), 529–542.

[ece370319-bib-0003] Asahina, S. (1991). Blattaria of Japan. Nakayama Shoten.

[ece370319-bib-0004] Austad, S. N. (1983). A game theoretical interpretation of male combat in the bowl and doily spider (*Frontinella pyramitela*). Animal Behaviour, 31(1), 59–73.

[ece370319-bib-0005] Djernæs, M. , & Varadinova, Z. (2020). Phylogeny and life history evolution of Blaberoidea (Blattodea) . Arthropod. https://arthropod‐systematics.arphahub.com/article/30129/

[ece370319-bib-0006] Durrant, K. L. , Skicko, I. M. , Sturrock, C. , & Mowles, S. L. (2016). Comparative morphological trade‐offs between pre‐ and post‐copulatory sexual selection in Giant hissing cockroaches (Tribe: Gromphadorhini). Scientific Reports, 6(1), 1–7. 27819321 10.1038/srep36755 PMC5098185

[ece370319-bib-0007] Enquist, M. , & Leimar, O. (1987). Evolution of fighting behaviour: The effect of variation in resource value. Journal of Theoretical Biology, 127(2), 187–205.

[ece370319-bib-0008] Friard, O. , & Gamba, M. (2016). BORIS: A free, versatile open‐source event‐logging software for video/audio coding and live observations. Methods in Ecology and Evolution/British Ecological Society, 7(11), 1325–1330.

[ece370319-bib-0009] Hsu, Y. , Earley, R. L. , & Wolf, L. L. (2005). Modulation of aggressive behaviour by fighting experience: Mechanisms and contest outcomes. Biological Reviews of the Cambridge Philosophical Society, 81(1), 33–74. 10.1017/S146479310500686X 16460581

[ece370319-bib-0010] Ito, H. , & Osawa, N. (2019). A field study of the colony composition of the wood‐feeding cockroach *Panesthia angustipennis spadica* (Blattodea: Blaberidae). Applied Entomology and Zoology, 54(1), 79–84.

[ece370319-bib-0011] Jormalainen, V. (1998). Precopulatory mate guarding in crustaceans: Male competitive strategy and intersexual conflict. The Quarterly Review of Biology, 73(3), 275–304.

[ece370319-bib-0012] Kojima, W. , & Lin, C. P. (2017). It takes two to tango: Functional roles, sexual selection and allometry of multiple male weapons in the flower beetle Dicronocephalus wallichii bourgoini. Biological Journal of the Linnean Society, 121(3), 514–529.

[ece370319-bib-0013] Kvarnemo, C. (2005). Evolution and maintenance of male care: Is increased paternity a neglected benefit of care? Behavioral Ecology, 17(1), 144–148.

[ece370319-bib-0014] Liu, P.‐C. , & Hao, D.‐J. (2019). Effect of variation in objective resource value on extreme male combat in a quasi‐gregarious species, Anastatus disparis. BMC Ecology, 19(1), 21. 31122223 10.1186/s12898-019-0237-9 PMC6533655

[ece370319-bib-0015] Maekawa, K. , Matsumoto, T. , & Nalepa, C. A. (2008). Social biology of the wood‐feeding cockroach genus *Salganea* (Dictyoptera, Blaberidae, Panesthiinae): Did ovoviviparity prevent the evolution of eusociality in the lineage? Insectes Sociaux, 55(2), 107–114.

[ece370319-bib-0016] McCann, T. S. (1981). Aggression and sexual activity of male southern elephant seals, *Mirounga leonina* . Journal of Zoology, 195(3), 295–310.

[ece370319-bib-0017] O'Neill, S. L. , Rose, H. A. , & Rugg, D. (1987). Social behaviour and its relationship to field distribution in Panesthia cribrata Saussure (Blattodea: Blaberidae). Australian Journal of Entomology, 26(4), 313–321.

[ece370319-bib-0018] Pandolfi, M. , Scaia, M. F. , & Fernandez, M. P. (2021). Sexual dimorphism in aggression: Sex‐specific fighting strategies across species. Frontiers in Behavioral Neuroscience, 15, 659615. 34262439 10.3389/fnbeh.2021.659615 PMC8273308

[ece370319-bib-0019] R Core Team . (2023). R: A language and environment for statistical computing. R Foundation for Statistical Computing. https://www.R‐project.org/

[ece370319-bib-0020] Royle, N. J. , Alonzo, S. H. , & Moore, A. J. (2016). Co‐evolution, conflict and complexity: What have we learned about the evolution of parental care behaviours? Current Opinion in Behavioral Sciences, 12, 30–36.

[ece370319-bib-0021] Smith, J. M. , & Price, G. R. (1973). The logic of animal conflict. Nature, 246(5427), 15–18.

[ece370319-bib-0022] Suzuki, S. (1999). Does carrion‐burial by Nicrophorus vespilloides (Silphidae: Coleoptera) prevent discovery by other burying beetles? 10.5555/19990506146

